# Squamous Cell Carcinoma Arising From an Epidermal Cyst of the Buttock: A Case Report

**Published:** 2019-10-21

**Authors:** Michika Fukui, Natsuko Kakudo, Naoki Morimoto, Masakatsu Hihara, Hiromu Masuoka, Kenji Kusumoto

**Affiliations:** Department of Plastic and Reconstructive Surgery, Kansai Medical University, Osaka, Japan

**Keywords:** squamous cell carcinoma, epidermal cyst, malignant transformation, large and long-standing tumor, rapid growth

## CASE DESCRIPTION

**Figure 1:**
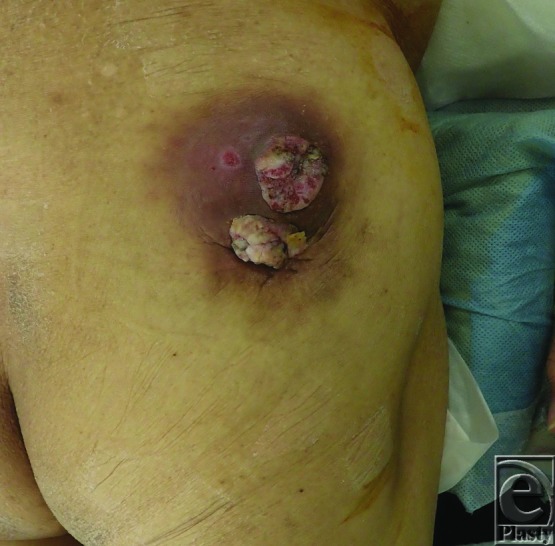
Preoperative findings. A 70-year-old man with a tumor (7×7 cm) on his buttock.

**Figure 2:**
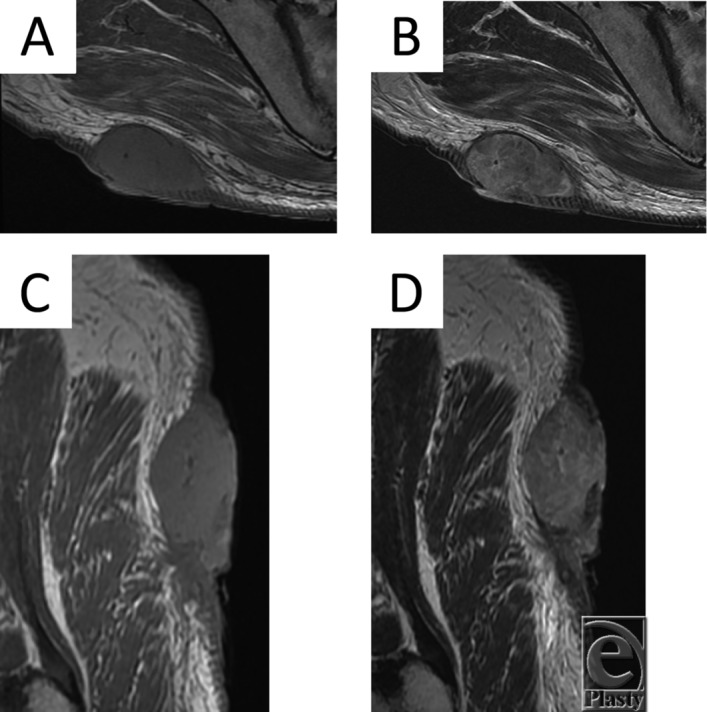
(*a*) Axial T1-weighted MRI scan of the tumor. (*b*) Axial T2-weighted MRI scan of the tumor. (*c*) Sagittal T1-weighted MRI scan of the tumor. (*d*) Sagittal T2-weighted MRI scan of the tumor.

**Figure 3:**
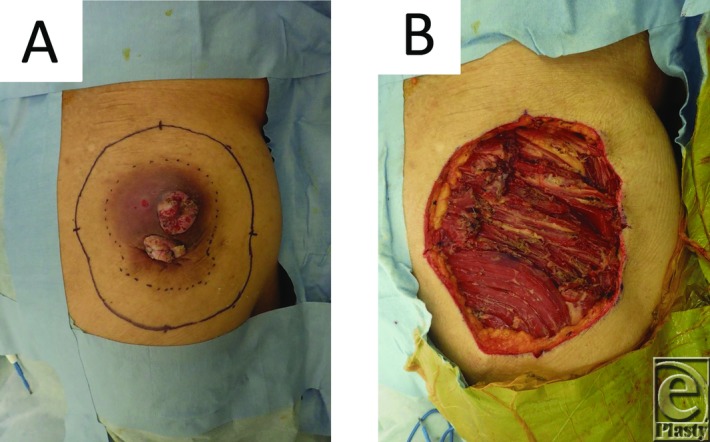
(*a*) Design of the operation. (*b*) Image obtained after the tumor was resected.

**Figure 4:**
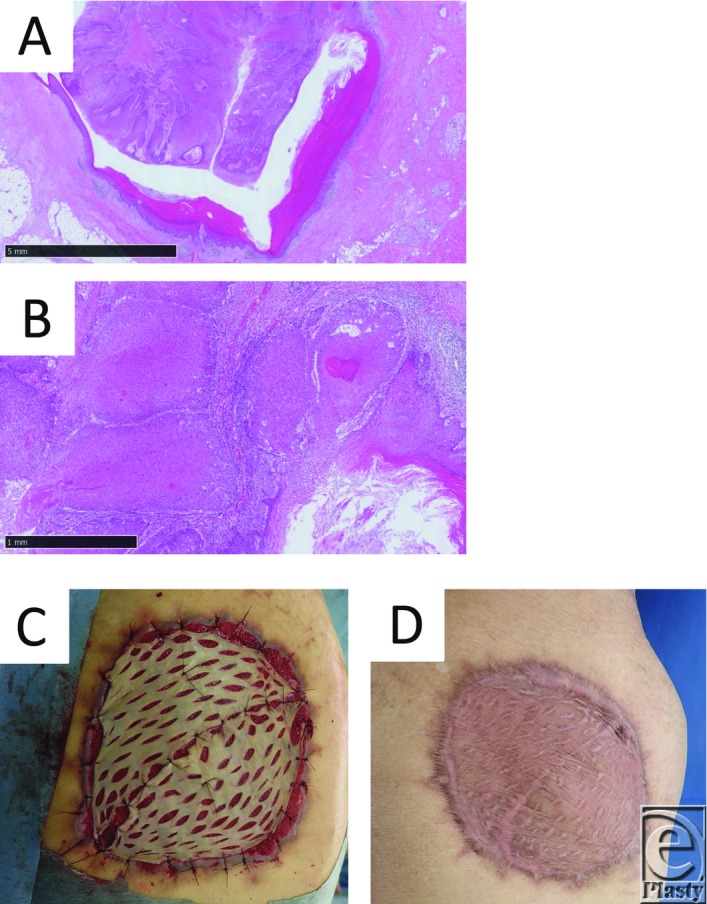
(*a*) Epithelial findings. The epithelium of the squamous cell carcinoma is contiguous with the epithelium of the epidermal cyst (hematoxylin and eosin staining, bar: 5 mm). (*b*) Cellular findings. Numerous keratinized atypical cells are seen. The atypical cells have formed nests and cancer pearls (hematoxylin and eosin staining, bar: 1 mm). (*c*) Postoperative findings obtained after the skin grafting. (*d*) Postoperative 6-month findings.

A 70-year-old man presented with a mass on his right buttock, which measured 7 × 7 cm in diameter (Fig 1), and a swelled right inguinal lymph node. He noticed the mass 20 years ago. It was infected and discharged pus, and recently it has been growing rapidly. Antibiotics were ineffective. Magnetic resonance imaging showed that it had invaded the subcutaneous region (Figs 2*a*-2*d*). We excised it with a 3-cm surgical margin and the muscle fascia, as well as the swelled lymph node (Figs 3*a* and 3*b*). Histopathologically, this case was diagnosed as a squamous cell carcinoma arising from an epidermal cyst and right inguinal lymph node metastasis, because the epithelium of the squamous cell carcinoma was contiguous with the epithelium of the epidermal cyst (Figs 4*a* and 4*b*). A skin graft and lymph node dissection were performed (Fig 4*c*). There was no recurrence of the tumor (Fig 4*d*).

## QUESTIONS

1. How often does squamous cell carcinoma arise from epidermal cysts?
2. What are the clinical symptoms of the malignant transformation of an epidermal cyst?
3. Why do some epidermal cysts become malignant?
4. How is the malignant transformation of an epidermal cyst diagnosed pathologically?

## DISCUSSION

Although epidermal cysts are the most common tumors affecting the body surface, the malignant transformation of epidermal cysts is very rare and very few cases of such transformation have been reported in the literature. The incidence of the malignant transformation of epidermal cysts ranges from 0.011% to 0.045%.^1^^-^4 In 2018, Franc et al^1^ reviewed 42 well-documented cases in which squamous cell carcinoma arose from an epidermal cyst. The mean age of these patients was 61.8 years (range, 28-96 years), with males affected more often than females (69% vs 31%). The head and the neck were the most commonly affected sites (54.8%). Some review articles showed that malignant transformation was common in middle-aged men and that the head and the neck were the most commonly affected regions.^2^^,^^3^^,^^5^ However, in Japanese reports the most commonly affected region was the buttocks,^6^ as was found in our case. In a review, Franc et al^1^ reported that the lesion size ranged from 0.7 to 20 cm (mean: 5.0 cm) and the lesions had been present for 0.5 to 480 months (mean: 92.6 months). The most common symptoms were pain (24.2%), rapid enlargement (48.6%), and overlying skin changes such as erythema or ulceration (38.2%), and antibiotic therapy failed prior to the excision of the lesion in 25% of cases.^1^

It has been suggested that the factors responsible for triggering malignant changes in epidermal cysts include chronic skin disease, actinic damage, and chronic irritation.^1^^,^^3^ In addition, human papillomavirus and trauma have been reported to cause such malignant changes.^2^^,^^5^^,^^7^ However, the mechanism underlying the malignant transformation of epidermal cysts remains unclear. Our case involved a long disease duration, so chronic irritation might have contributed to the malignant transformation of the epidermal cyst. In addition, our patient's medical history included infection, the discharge of pus, and inflammatory changes.

Normally, epidermal cysts range in size from 1 to 4 cm, and on MRI scan they typically present as well-defined oval lesions, which exhibit intermediate to slightly increased signal intensity on T1-weighted images and high signal intensity on T2-weighted/fluid-sensitive sequences.^8^ However, ruptured cysts frequently exhibit non-specific MRI signal characteristics.^8^ The MRI findings of the present case differed from those of typical epidermal cysts and the lesion was a solid tumor, rather than a cystic lesion. Clinically, squamous cell carcinoma arising from an epidermal cyst should be actively suspected in cases involving large, long-standing, rapidly growing, dark red tumors, in which (1) the tumor is accompanied by small ulcers, (2) prominent scarred nodules are present on the tumor, (3) the tumor exhibits atypical MRI findings, and (4) the tumor displays symptoms of an antibiotic-resistant infection. Therefore, when antibiotic treatment is not effective in such cases, malignant transformation should be suspected.

The following 4 items can be considered to be clinical and histopathological evidence of the malignant transformation of an epidermal cyst^6^: (1) the epidermal cyst is long-standing and later grows rapidly; (2) the tumor has the organizational structure of a keratinous cyst, which is similar to the structure of an epidermal cyst; (3) a part of the tumor is not malignant; and (4) the nonmalignant part of the tumor is contiguous with the region of atypical cell proliferation. Since the present case met the criteria for (1), (3), and (4), it was diagnosed as a squamous cell carcinoma that arose from an epidermal cyst. Moreover, we suggest that intermittent infection (inflammatory stimulation) and rupturing of the cyst (mechanical stimulation) might have contributed to the malignant transformation of the cyst.^1^ Epidermal cysts are encountered in routine clinical practice, and it should be noted that when infected epidermal cysts are left (not excised) for a long time, they can transform into malignant lesions.

## SUMMARY

We report a case in which squamous cell carcinoma arose from an epidermal cyst on the patient's buttock. Clinically, squamous cell carcinoma arising from an epidermal cyst should be actively suspected in cases in which a large, long-standing, rapidly growing epidermal cyst is accompanied by ulceration and prominent scarred nodules and exhibits atypical MRI findings and symptoms of an antibiotic-resistant infection.

## References

[1] Frank E, Macias D, Hondrop B, Kerstetter J, Inman JC (2018). Incidental squamous cell carcinoma in an epidermal inclusion cyst. Case Rep Dermatol.

[2] Sze S, Richmond I, Bickers A, Saha A (2016). Squamous cell carcinoma arising from a vulval epidermal cyst. J Obstet Gynaecol Res.

[3] Sakamoto A, Shiba E, Hisaoka M (2015). Squamous cell carcinoma arising from an epidermal cyst in the thumb. Int J Surg Case Rep.

[4] Yeh L-P, Liao K-S (2013). Squamous cell carcinoma arising from an epidermal cyst of the scrotum. Tzu Chi Med J.

[5] Lee JW, Shin JY, Roh SG, Lee NH, Yang KM (2016). Squamous cell carcinoma arising from an epidermal inclusion cyst. Arch Plast Surg.

[6] Suzuki M, Hashimoto K (2017). A case of squamous cell carcinoma arising in atheroma of the perineum. Ymaguchi Igaku.

[7] Skroza N, Proietti I, Tolino E (2014). Isotretinoin for the treatment of squamous cell carcinoma arising in an epidermal cyst. Dermatol Ther.

[8] Houdek MT, Warneke JA, Pollard CM, Lindgren EA, Taljanovic MS (2010). Giant epidermal cyst of the gluteal region. Radiol Case Rep.

